# Real Time Enzyme Inhibition Assays Provide Insights into Differences in Binding of Neuraminidase Inhibitors to Wild Type and Mutant Influenza Viruses

**DOI:** 10.1371/journal.pone.0023627

**Published:** 2011-08-17

**Authors:** Susan Barrett, Peter G. Mohr, Peter M. Schmidt, Jennifer L. McKimm-Breschkin

**Affiliations:** 1 CSIRO Materials Science and Engineering, Parkville, Australia; 2 CSIRO Australian Animal Health Laboratory, Geelong, Australia; University of Georgia, United States of America

## Abstract

The influenza neuraminidase (NA) inhibitors zanamivir, oseltamivir and peramivir were all designed based on the knowledge that the transition state analogue of the cleaved sialic acid, 2-deoxy,2,3-dehydro N-acetyl neuraminic acid (DANA) was a weak inhibitor of NA. While DANA bound rapidly to the NA, modifications leading to the improved potency of these new inhibitors also conferred a time dependent or slow binding phenotype. Many mutations in the NA leading to decreased susceptibility result in loss of slow binding, hence this is a phenotypic marker of many but not all resistant NAs. We present here a simplified approach to determine whether an inhibitor is fast or slow binding by extending the endpoint fluorescent enzyme inhibition assay to a real time assay and monitoring the changes in IC_50_s with time. We carried out two reactions, one with a 30 min preincubation with inhibitor and the second without. The enzymatic reaction was started via addition of substrate and IC_50_s were calculated after each 10 min interval up to 60 min. Results showed that without preincubation IC_50_s for the wild type viruses started high and although they decreased continuously over the 60 min reaction time the final IC_50_s remained higher than for pre-incubated samples. These results indicate a slow equilibrium of association and dissociation and are consistent with slow binding of the inhibitors. In contrast, for viruses with decreased susceptibility, preincubation had minimal effect on the IC_50_s, consistent with fast binding. Therefore this modified assay provides additional phenotypic information about the rate of inhibitor binding in addition to the IC_50_, and critically demonstrates the differential effect of incubation times on the IC_50_ and *K*_i_ values of wild type and mutant viruses for each of the inhibitors.

## Introduction

Two licensed neuraminidase (NA) inhibitors (NAIs) are currently approved globally for the treatment and prevention of influenza, zanamivir (Relenza™) and oseltamivir (Tamiflu™). A third compound peramivir has recently received approval in Japan and had emergency authorisation for limited use during the pandemic outbreak [1]. All compounds were designed based on the knowledge of the structure of sialic acid bound in the NA active site [2]. The transition state analogue of sialic acid, 2-deoxy,2,3-dehydro N-acetyl neuraminic acid (DANA) was known to be a weak inhibitor of the NA. Addition of an amino group at the 4-position of DANA led to around 100-fold enhancement of the inhibitory activity whereas the addition of a guanidinium group (zanamivir) led to around a 10,000-fold enhancement [3]. Addition of the guanidinium group led to zanamivir being a time dependent, or slow binding inhibitor [3], [4]. The hypothesis for the slow binding of zanamivir is that a water molecule has to be displaced before the guanidinium group can bind tightly in the active site [4]. While oseltamivir is also a slow binding inhibitor, this is thought to be due to the need for the rotation of the E276 in the enzyme active site [5] to accommodate binding of its hydrophobic side chain [6]–[8]. Peramivir contains both the guanidinium group as in zanamivir, and a hydrophobic side chain as in oseltamivir. Hence it is also a slow binding inhibitor possibly impacted by both mechanisms [6]. Some NAs with mutations conferring resistance to the NAIs appear to have lost this slow binding phenotype [6], [8]–[11]. Thus in addition to an increase in IC_50_, loss of slow binding can also be a phenotypic marker of reduced susceptibility.

Sensitivity to influenza NAIs is determined by two types of enzyme inhibition assays, a fluorescent based assay which uses 4-Methylumbelliferyl N-acetyl-α-D-neuraminic acid (MUNANA) [12] and a chemiluminescent assay based on the NA-Star substrate [13], [14]. The inhibition assay includes preincubation of NA with its inhibitor, initiation of the enzymatic reaction by addition of substrate, and finally addition of a high pH solution which stops the reaction, and enhances the fluorescent or chemiluminescent signal. Protocols for the fluorescent assay vary between different laboratories for the preincubation times and temperatures, assay incubation time and buffers used, all of which can impact on the IC_50_ [14]. Hence there is a need for a standardized assay to enable comparisons of results between different laboratories. There has been no study of how incubation times affect IC_50_s, although Pegg et al. [4] reported that for binding of zanamivir to an N2 NA the apparent *K*_i_ varied by 10,000-fold depending on the incubation conditions. The availability of more sensitive fluorimeters with kinetics functions means we can continuously monitor changes in enzyme activity and therefore changes in IC_50_ with time. We have now modified the basic MUNANA assay, to a real time assay, and have developed what we term IC_50_ kinetics assays. This expands the information obtained from inhibition assays to also provide information about the slow or fast binding phenotype of an NA [11]. Thus this approach provides additional information about potential NAI resistance and the impacts of mutations on inhibitor binding. The chemiluminescent assay, commercialised as NA-Star® (Applied Biosystems), is a rapid reaction, with a substrate half life of around 15 min, and a very low signal strength necessitating the addition of an enhancer. Hence this assay currently does not lend itself to real time analysis. Our aim was to investigate whether our IC_50_ kinetics assay could distinguish differences in binding of the NAIs to a panel of wild type and known mutants with decreased susceptibility. Our results demonstrate that this assay can readily distinguish fast and slow binding of the NAIs, and among the viruses tested the mutant viruses all demonstrated loss of slow binding compared to the parent wild type virus.

## Methods

### Viruses and inhibitors

The wild type and mutant viruses used were: A/Mississippi/03/01 H1N1 wild type and H274Y mutant with decreased susceptibility to oseltamivir [15], A/Fukui/45/04 H3N2 wild type and E119V mutant with decreased susceptibility to oseltamivir [16], B/Perth/211/01 influenza B wild type and D197E mutant with decreased susceptibility to all NAIs [17], NWS/G70C H1N9, a reassortant with the HA of NWS and NA from A/tern/Australia/G70C/75, wild type and E119G mutant with decreased susceptibility to zanamivir and peramivir [9], [18] and NWS/G70C R292K mutant with decreased susceptibility to all the NAIs [10]. As an additional comparison for how different N1 NAs behaved in the assays, we also used an egg cultured and gamma-irradiated H1N1 pandemic influenza virus (A/Swine/Shepparton/2009). The original sample for this virus was submitted by the Department of Primary Industries, Victoria, Australia to the CSIRO, Australian Animal Health Laboratory, Diagnostic, Surveillance and Response group where the virus was cultured. The N1 NAs of two gamma-irradiated H5N1 viruses were also assayed from a clade 1 H5N1 virus from Vietnam, and a clade 2 H5N1 virus from Indonesia [19]. Both clades were included since we have previously shown that the clade 2 Indonesian viruses had a higher IC_50_ for oseltamivir compared to the clade 1 viruses, so we wanted to see if this was reflected by any differences in the binding kinetics. DANA was purchased from Sigma (Australia), zanamivir and peramivir were synthesised by GlaxoSmithKline (Stevenage, UK) and oseltamivir carboxylate was produced from oseltamivir phosphate by Dr Keith Watson (Walter and Eliza Hall Institute, Australia). Dilutions of inhibitors were prepared in water, ranging from 0.001 nM to 100,000 nM and 7-serial log_10_ dilutions were used, the concentrations depended on the wild type or mutant virus assayed.

### Enzyme assays

#### NA inhibition assays

Viruses were solubilised and inactivated by the addition of CHAPS (3-[(3-Cholamidopropyl)dimethylammonio]-1-propanesulfonate) to a final concentration of 1%. We used the fluorescent MUNANA-based assay for all experiments [12]. Enzyme activity was titrated for all viruses to ensure linearity of the enzymatic reaction over time. Assays contained 50 µl of virus or 25 µl of virus+25 µl of inhibitor and 50 µl of 200 µM MUNANA (Carbosynth Berkshire, UK) diluted in 100 mM sodium acetate pH 5.5 and 10 mM CaCl_2_. We used a BMG FLUOstar Optima reader and the kinetics function for real time monitoring of the fluorescent signal. Inhibition assays were performed in two ways. The first method used a 30 min preincubation of viruses with serial dilutions of inhibitors followed by the addition of MUNANA substrate [9]. The second method added serial dilutions of inhibitors and MUNANA simultaneously to the NA omitting the pre-incubation step. Fluorescence for both assays was monitored at 1 min intervals for 60 min after addition of substrate, to ensure there were no significant fluctuations between the 10 min data points. Graphs of concentration of inhibitor versus percent enzyme inhibition compared to the control were plotted after 10, 20, 30, 40, 50 and 60 min. The IC_50_ was calculated as the concentration of inhibitor resulting in a 50% reduction in fluorescent units (FU) compared to the control. IC_50_s were then plotted as bar graphs for each of the time points for both assays. Alternatively IC_50_s were also calculated based on the difference in FU between consecutive 10 min time points, i.e. 10–20, 20–30, 30–40, 40–50 and 50–60 min and the relative rates were plotted against the drug concentrations. The IC_50_ was the drug concentration leading to 50% inhibition of the rate of reaction compared to the control rate.

### *K*_m_ and *K*_i_

To calculate the Michaelis Menten constant *K*_m_, hydrolysis of MUNANA was measured at substrate concentrations ranging from 6.25 to 200 µM, with readings taken every minute in the BMG FLUOstar Optima reader. Experiments were carried out in duplicate and repeated twice. The maximum slope for each reaction was determined by comparing the slopes over different overlapping time intervals. Initial velocities of the reactions were then calculated by measuring the maximum slopes plotted as a function of substrate concentrations. The *K*_m_ was calculated using a nonlinear regression function in GraphPad Prism. Setting the constraints of the *K*_m_ and the substrate concentration at 100 µM, the *K*_i_ was calculated using nonlinear regression and one-site competitive binding in GraphPad Prism [20].

## Results

### Kinetics of inhibitor binding

**Figure 1:**
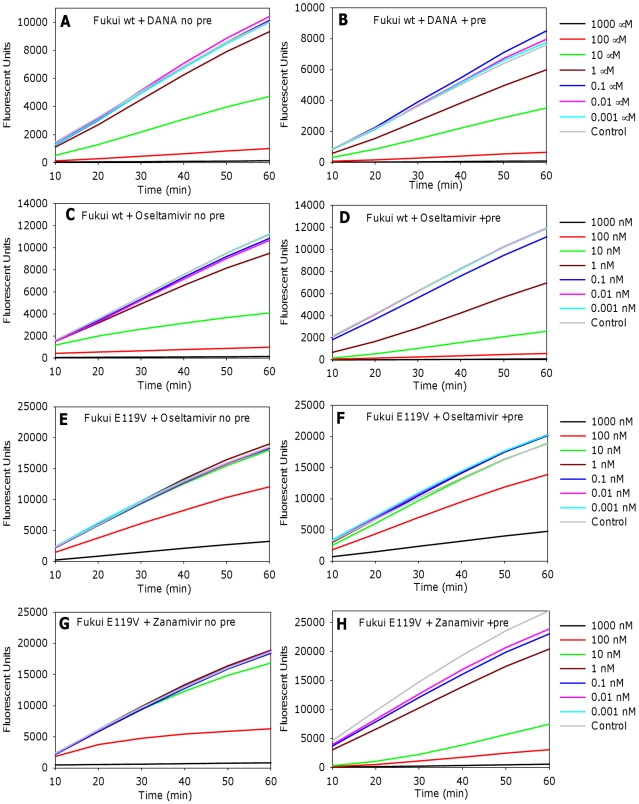
Comparison of the effects of preincubation or no preincubation with inhibitor on the enzyme activity. A/Fukui/45/04 H3N2 wild type virus and E119V mutant virus with reduced oseltamivir susceptibility were incubated in serial dilutions of inhibitors, either with or without preincubation, to compare the effects on the reaction rates. Left panels no preincubation, right panels 30 min preincubation. (A, B) wild type with DANA, (C, D) wild type with oseltamivir, (E, F) E119V with oseltamivir, (G, H) E119V with zanamivir. The curves for both reactions for DANA (A, B), and for E119V with oseltamivir (E, F) show a fairly constant reaction rate, and correlate with fast binding. The differences in the curves with and without preincubation for oseltamivir binding to wild type virus (C, D) and for zanamivir binding to the E119V mutant (G, H) and the decrease in rates during the reactions without preincubation, (C, G), are all consistent with a slow binding phenotype.

If an inhibitor is time dependent/slow binding, then preincubation of NA in the absence of substrate should increase the percentage of occupied substrate binding sites, leading to lower initial IC_50_ values. Conversely a fast binding inhibitor should quickly reach equilibrium reducing the impact of preincubation to a minimum. Wild type and mutant viruses were either preincubated with 10-fold dilutions of inhibitors for 30 min prior to the addition of substrate or inhibitors and substrate were added simultaneously to the virus. Both reactions were continuously monitored for 60 min. We used DANA as our model for a fast binding NAI to validate our hypothesis that there should be no difference with or without preincubation for fast binding NA inhibitors. Results confirmed that preincubation with DANA did not affect the relative reaction rates for any of the wild type or mutant viruses. Figures 1A and B show graphs of NA activity for the 60 min reactions for A/Fukui/45/04 H3N2 wild type and DANA.

In contrast to DANA, NA activity curves of viruses incubated with oseltamivir, zanamivir and peramivir varied between no preincubation and preincubation reactions, depending on whether the virus was sensitive or had reduced susceptibility to each inhibitor. To demonstrate the differences in the types of curves we observed results for A/Fukui/45/04 H3N2 wild type and E119V mutant, with reduced oseltamivir susceptibility, are shown in Figure 1C-H for oseltamivir and zanamivir.

With increasing drug concentrations differences in the shapes of the curves were noted. Where the target virus was sensitive to the respective inhibitor, (wild type virus and oseltamivir C, D; E119V and zanamivir, G, H) the curves showed a decrease in rate with no preincubation, consistent with the inhibitors binding slowly, leading to increased inhibition with time. These results are similar to those originally reported by Pegg et al. in 1994 [21]. In contrast, after preincubation with inhibitor and addition of substrate, there was a gradual increase in rate, suggesting some dissociation of inhibitor. Reaction curves for the E119V mutant virus incubated with oseltamivir were similar either with or without preincubation, similar to what we observed with DANA (Figure 1E, F). This is consistent with rapid binding of oseltamivir to the E119V mutant.

**Figure 2:**
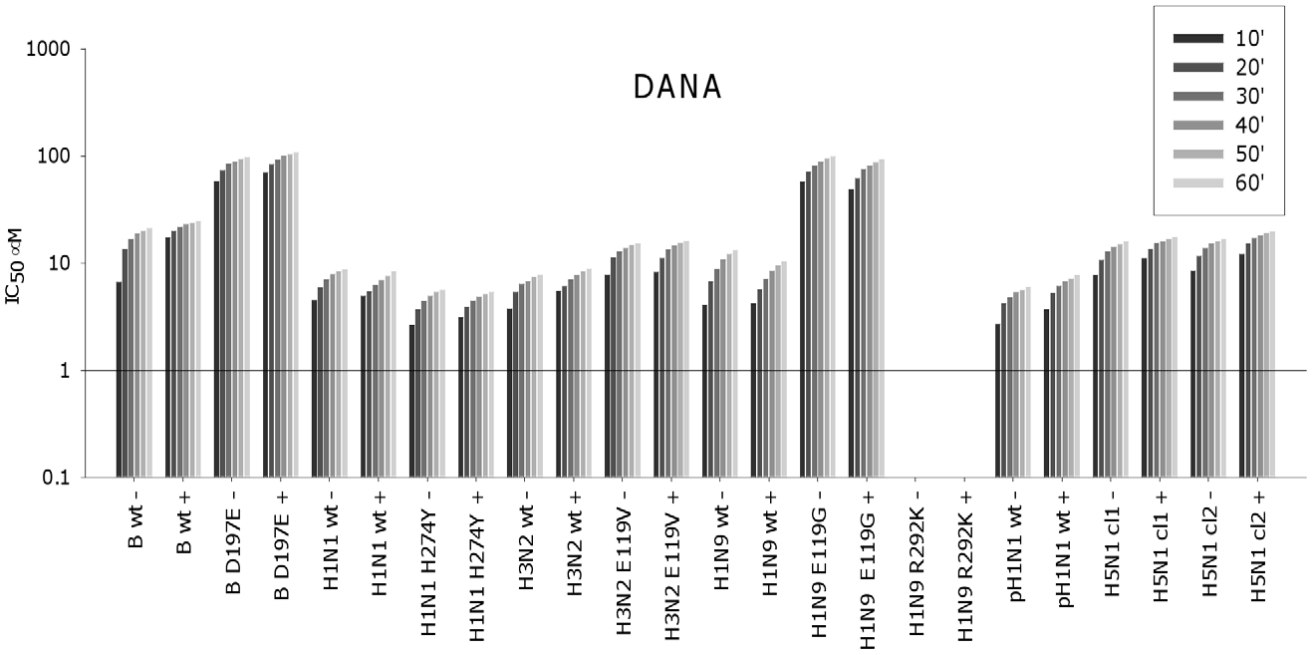
Effect of incubation time on IC_50_s for DANA. Wild type and mutant viruses were preincubated for 30 min with DANA, or virus, DANA and substrate were co-incubated without a preincubation step. IC_50_s were calculated after each 10 min up to 60 min, after the addition of the MUNANA substrate. The IC_50_s were similar with (+) or without (−) preincubation, correlating with fast binding. Results are the mean of duplicate assays. Virus abbreviations, wt = wild type, mutants have specific mutation defined. B = B/Perth/211/01, H1N1 = A/Mississippi/03/01, H3N2 = A/Fukui/45/04, H1N9 = A/NWS/tern/Australia/G70C/75, pH1N1 = pandemic H1N1 A/Swine/Shepparton/2009, H5N1 cl1 = Vietnam clade 1 H5N1, H5N1 cl2 = Indonesian clade 2 H5N1.

**Figure 3:**
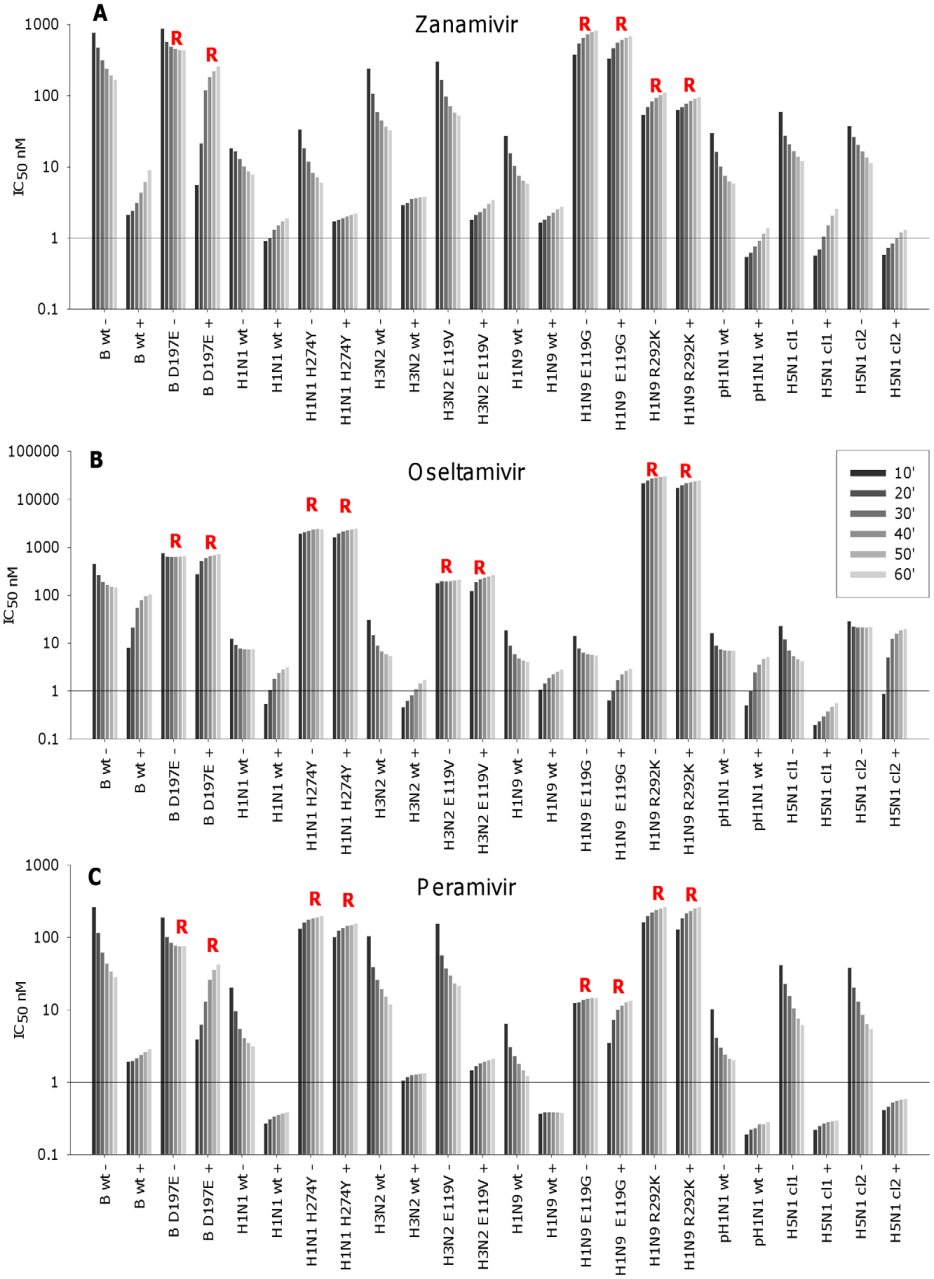
Effect of incubation time on IC_50_s for zanamivir, oseltamivir and peramivir. Wild type and mutant viruses were preincubated for 30 min with inhibitor (+) before addition of substrate, or virus, inhibitor and substrate were all co-incubated without a preincubation step (−). IC_50_s were calculated after addition of substrate for each 10 min up to 60 min. (A) Final higher IC_50_ values for binding of zanamivir without preincubation compared to preincubation and the decreases in IC_50_s without preincubation over the 60 min correlate with slow binding. For D197E, E119G and R292K final 60 min values are similar for both reactions, which indicate loss of slow binding and correlates with reduced susceptibility to zanamivir. (B) For oseltamivir final higher IC_50_ values for reactions without preincubation compared to preincubation and decreases in IC_50_s without preincubation indicate slow binding to wild type viruses. For D197E, H274Y, E119V, R292K final 60 min values are similar which indicates loss of slow binding and correlates with reduced susceptibility to oseltamivir. (C) For peramivir final higher IC_50_ values for reactions without preincubation compared to preincubation, and decreases in IC_50_s without preincubation indicate slow binding. For D197E, H274Y, E119G and R292K final 60 min values are similar for both reactions, which indicate the loss of slow binding and correlates with reduced susceptibility to peramivir. Results are means of duplicate assays. R = reduced susceptibility. Virus abbreviations, wt = wild type, mutants have specific mutation defined. B = B/Perth/211/01, H1N1 = A/Mississippi/03/01, H3N2 = A/Fukui/45/04, H1N9 = A/ /NWS/ Tern Australia/ /70, pH1N1 = pandemic H1N1 A/Swine/Shepparton/2009, H5N1 cl1 = Vietnam clade 1 H5N1, H5N1 cl2 = Indonesian clade 2 H5N1.

To examine how these changes in catalysis are reflected in the respective IC_50_s over the reaction period, the IC_50_s were calculated for consecutive 10 min intervals for DANA, zanamivir, oseltamivir and peramivir against our panel of wild type and mutant viruses. Results are shown for each drug and virus combination in Figures 2 and 3 over the 60 min reaction period.

### IC50 kinetics of DANA

For DANA, preincubation with inhibitor did not increase binding efficacy compared to no preincubation, indicated by the similar IC_50_ values in Figure 2 for both reactions with each of the viruses. Both methods indicated maximum binding occurred rapidly, surprisingly with an increase in the IC_50_s over the 60 min incubation period in both assays. Some reduced susceptibility to DANA was seen with the B/Perth/211/01 D197E virus compared to the wild type virus. This mutation affects the interaction of R152 with the N-acetyl group on the sugar ring [11] conferring reduced susceptibility to all inhibitors. DANA also showed reduced binding to the E119G mutant NA, presumably due to loss of hydrogen bonding between the 4′-OH and the COOH on the E119. Binding to the R292K mutant NA was not tested for DANA since this mutation also affects binding of all inhibitors through altered interactions with the C2 COOH group [5], and would have required concentrations of around 1 mM DANA. Although blank, this has been left in the graphs to enable alignment of all the graphs in Figures 2 and 3.

### IC_50_ kinetics of zanamivir

For the viruses known to be sensitive to zanamivir, a 30 min preincubation step clearly resulted in lower IC_50_s compared to no preincubation (Figure 3A). Furthermore, there was a gradual decrease in IC_50_ over 60 min without the preincubation step. Both results are consistent with slow binding. In contrast, for viruses with reduced susceptibility to zanamivir preincubation did not enhance binding, since there was very little difference in the final zanamivir IC_50_s between the two reactions (Table 1). Without preincubation the IC_50_s for B/Perth/211/01 D197E plateaued rapidly, or for the NWS/G70C E119G and R292K they increased. This is consistent with the loss of slow binding.

**Table 1:** Comparison of final IC_50_ (nM) values of reactions with and without preincubation after 60 min^a^.

	^b^B/Perth wt	B/Perth D197E	Miss'pi H1N1 wt	Miss'pi H1N1 H274Y	Fukui H3N2 wt	Fukui H3N2 E119V	5H1N9 wt	G70C H1N9 E119G	G70C H1N9 R292K	Swine pH1N1	Avian H5N1 Clade1	Avian H5N1 Clade2
**Zanamivir** No pre	167	434	7.8	6.0	32.4	51.9	5.7	816	110	5.8	12.0	11.2
30′ pre	8.9	258	1.9	2.2	3.8	3.4	2.7	678	94.8	1.4	2.5	1.3
**No pre/pre**	**18.8**	**1.7**	**4.1**	**2.7**	**8.5**	**15.3**	**2.1**	**1.2**	**1.2**	**4.2**	**4.7**	**8.6**
**Oseltamivir** No pre	144	660	7.3	2353	5.3	208	4.0	5.4	30139	6.8	4.1	21.4
30′ pre	104	708	3.1	2440	1.7	260	2.8	2.9	24692	5.1	0.6	19.6
**No pre/pre**	**1.4**	**0.9**	**2.4**	**1.0**	**3.2**	**0.8**	**1.5**	**1.9**	**1.2**	**1.3**	**7.4**	**1.1**
**Peramivir** No pre	27.8	75.2	3.1	196	11.9	21.4	1.2	14.6	261	2.0	6.2	5.4
30′ pre	2.8	41.5	0.4	153	1.3	2.1	0.4	13.3	260	0.3	0.3	0.6
**No pre/pre**	**9.8**	**1.8**	**8.2**	**1.3**	**9.0**	**10.2**	**3.2**	**1.1**	**1.0**	**7.1**	**21.0**	**9.3**

a IC_50_ values are the average of duplicate assays.

Viruses, wt = wild type, mutants have specific mutation defined. B/Perth/211/01, A/Mississippi/03/01, A/Fukui/45/04, A/NWS/tern/Australia/G70C/75, pandemic H1N1 A/Swine/Shepparton/2009, Vietnam clade 1 avian H5N1, Indonesian clade 2 avian H5N1.

There was an increase in IC_50_ for all viruses as soon as substrate was added after preincubation with zanamivir, indicating some dissociation of inhibitor. The amount of dissociation varied among the different wild type viruses. IC_50_s increased over the 60 min by more than four-fold for the wild type B/Perth/211/01 and Clade 1 H5N1 viruses compared to two-fold or less for many of the other viruses (Table S1).

### IC_50_ kinetics of oseltamivir

Most viruses sensitive to oseltamivir demonstrated a decrease in IC_50_ without preincubation during the 60 min reaction (Figure 3B). The IC_50_s were lower after preincubation, although interestingly the differences in the final IC_50_s with and without preincubation were mostly less than seen for the binding of zanamivir and peramivir, (Table 1). For those viruses with known reduced susceptibility to oseltamivir, the B/Perth/211/01 D197E, A/Mississippi/03/01 H274Y, A/Fukui/45/04 E119V, and NWS/G70C R292K, there was clearly loss of slow binding since preincubation had minimal effect on decreasing the IC_50_s (Figure 3B). Interestingly the reactions discriminated differences in the apparent dissociation rates for the different inhibitors. Comparisons of the increases in IC_50_ between 10 and 60 min after preincubation showed that in general there was a greater fold increase for oseltamivir for wild type viruses, compared to increases seen with zanamivir. Increases with zanamivir were greater than those seen for peramivir (Table S1). Three wild type viruses (B/Perth/211/01, Indonesian Clade 2 H5N1 and A/Swine/Shepparton/ 2009 H1N1) had an intermediate phenotype for dissociation of oseltamivir. The final IC_50_s with or without preincubation were similar (Table 1), indicating they were not slow binding compared to other wild type viruses. However, the changes in IC_50_s over the 60 min time course were different to the pattern seen with the more resistant mutant viruses (>20-fold reduced susceptibility). After preincubation the IC_50_s of these mutant viruses were already high and there were only small increases in the IC_50_s over 60 min. In contrast, these three wild type viruses had an initial lower IC_50_ after preincubation, but there was more than a 10-fold increase in IC_50_ over the 60 min reaction time (Table S1). Therefore by following the changes over time we have identified differences in the apparent dissociation which would not have been obvious from the standard single end point IC_50_s.

### IC_50_ kinetics of peramivir

For all the wild type viruses preincubation with peramivir enhanced inhibition (Figure 3C). IC_50_s for most wild type viruses were more than 8-fold lower than without preincubation (Table 1). Decreases in IC_50_s were seen without preincubation during the 60 min reaction. For most wild type viruses without preincubation there was a greater change in IC_50_ during the 60 min reaction for peramivir than zanamivir, with the least change seen in the oseltamivir reactions (Table S1). This is the reverse seen for the preincubation reaction, and would relate to the very slow binding of peramivir compared to the other two inhibitors [22]–[24]. We also saw loss of slow binding with those viruses with reduced susceptibility to peramivir, which include the B/Perth/211/01 D197E, the A/Mississippi/03/01 H274Y, the NWS/G70C E119G, and R292K. As seen for the B/Perth/211/01 D197E with the other inhibitors, without preincubation there was a rapid plateau in IC_50_s. For the other mutant viruses some dissociation was seen in the reactions without preincubation.

### Calculation of IC_50_ based on the rate of reaction

Traditionally the IC_50_ is calculated based on the relative FU for the control and drug treated samples at the end of a 45–60 min reaction period. As the IC_50_ is calculated based on the total FU, it actually represents the average enzyme inhibition up to that time point. This approach assumes the percent inhibition, and thus IC_50_ is constant for the entire period.

However, our results have shown when inhibitors are slow binding the reaction rates change more during the early parts of the reaction, but may stabilize and become more constant later in the reaction. Therefore, the analysis of IC_50_s based on reaction rates in separate time intervals, where each IC_50_ is independent of the preceding interval, might represent a more appropriate way to investigate the potency of an NAI. Rates were calculated based on the difference in FU between each 10 min. IC_50_s were calculated as the drug concentration causing 50% inhibition of the rate of the control reaction. In Table 2 we show how the IC_50_s compare based on the FU method or the rate method for the A/Mississippi/03/01 and A/Fukui/45/04 wild type viruses. With both methods the IC_50_s changes during the reaction, and there is up to a two-fold difference between the two methods after 60 min. The IC_50_s based on cumulative FU are all higher than the IC_50_s based on rates without preincubation and lower in the preincubation assays. As our data indicates that the reaction becomes more stable in the final intervals, the IC_50_s calculated for the last interval may be more accurate than the IC_50_ based on total FU, since it is not using the data from the preceding intervals.

**Table 2:** Differences in IC_50_ (nM) calculated on relative slope between time intervals^a^ versus total fluorescent units^b^ for A/Mississippi/03/01 and A/Fukui/45/04 wild type viruses.

	A/Mississippi/03/01					A/Fukui/45/04				
FU time point	0–20	0–30	0–40	0–50	0–60	0–20	0–30	0–40	0–50	0–60
Zanamivir no pre	16.4	12.8	10.1	8.6	7.8	106.0	58.6	44.4	36.7	32.4
Zanamivir pre	1.0	1.3	1.5	1.7	1.9	3.1	3.5	3.6	3.7	3.8
Oseltamivir no pre	9.1	7.6	7.4	7.2	7.3	14.5	8.8	6.6	5.8	5.3
Oseltamivir pre	1.0	1.8	2.4	2.8	3.1	0.6	0.8	1.1	1.4	1.7

Rates time interval	10–20	20–30	30–40	40–50	50–60	10–20	20–30	30–40	40–50	50–60
Zanamivir no pre	16.0	8.3	6.0	5.5	5.0	47.5	54.3	25.3	19.7	17.3
Zanamivir pre	1.1	1.8	2.3	2.6	3.4	3.2	4.4	3.8	4.2	4.5
Oseltamivir no pre	7.5	6.2	6.7	6.8	7.9	7.2	4.7	3.8	3.8	3.4
Oseltamivir pre	2.3	3.9	4.6	5.0	5.6	0.9	1.7	2.5	3.3	3.4

a IC_50_s for FU are based on the drug concentration resulting in 50% inhibition of the uninhibited control FU.

b Rates in FU/min are calculated for each drug concentration for each time interval and the IC_50_ is the drug concentration which inhibits the rate of the uninhibited control by 50%. This approach separates each reaction time so it is independent of the rates of preceding time intervals.

However, this means kinetics assays would have to be used in order to calculate the rates between time intervals. Hence while potentially more accurate for slow binding inhibitors, this approach is more time consuming for routine testing of large numbers of isolates, when compared to calculating IC_50_s based on the final FU value.

### Effect of reaction time on *K*_i_

**Figure 4:**
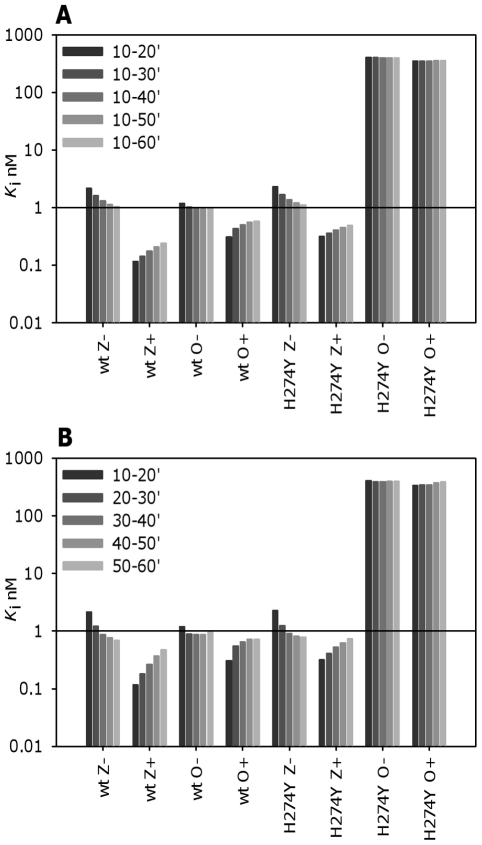
Effect of incubation times on *K*_i_ values for A/Mississippi/03/01 H1N1 wild type and H274Y mutant viruses. *K*_i_ values were calculated either (A) for the cumulative reaction time or (B) for each successive 10 min interval using the relative reaction rates in GraphPad Prism. For viruses sensitive to an inhibitor the *K*_i_ values changed over the course of the reaction. In contrast, for the H274Y virus binding to oseltamivir to which it has reduced susceptibility, the *K*_i_ stabilized more rapidly. Since the *K*_i_s in (A) are calculated based on the average rates there is less change than in (B) where each interval *K*_i_ is independent of the preceding rates. Hence in addition to the incubation time having a differential effect on the *K*_i_ values for wild type and mutant viruses, the time range used for the *K*_i_ calculations also affects the value. Results are means of duplicate assays. Z-zanamivir, O –oseltamivir (+) preincubation with inhibitor, (−) no preincubation.

Other groups also use the *K*_i_ as it is thought not to vary as much as the IC_50_ with different reaction conditions, namely the substrate concentration. We used two approaches to calculate the *K*_i_; the first calculated *K*_i_s for cumulative times (10–20′, 10–30′ etc.), which is similar to single time points used by others, (e.g. a 45 or 60 min reaction) and the second calculated *K*_i_s for each consecutive 10 min time interval (10–20′, 20–30′ etc.). The *K*_m_ values were calculated for the A/Mississippi/03/01 wild type (15 µM) and H274Y mutant (27.6 µM), and these were used to calculate the *K*_i_ values in GraphPad Prism. Results are shown in Figure 4A and B. For the mutant virus the *K*_i_ values for oseltamivir changed little over the course of the reaction with or without preincubation. For wild type viruses these *K*_i_ values changed with time in both methods with or without preincubation. This is not surprising since the reaction rates were constantly changing (exemplified by A/Fukui/45/04 in Figure 1), and the *K*_i_ depends on the reaction rate for each time interval. For oseltamivir and the wild type virus there was little change in the *K*_i_ after 40 min. For zanamivir the *K*_i_s continued to change through the 60 min period, indicating that the steady state had not yet been reached. The changes in *K*_i_ were less for the cumulative method since this averages the rates up to that time, whereas the *K*_i_s for consecutive time intervals are independent of the rates for preceding times. This data shows that although *K*_i_ is considered more invariant with regard to assay conditions it is affected in the same way as IC_50_s by the changing reaction rate over time.

## Discussion

We have shown here that a simple extension of the standard NA enzyme inhibition assay, to a real time IC_50_ kinetics assay provides an insight into whether mutations affect the kinetics of drug binding, without the need for complicated equations. The assay can be carried out in any laboratory with a filter based fluorimeter, and kinetics programs, hence requires no additional equipment or software. By comparing the assay with and without preincubation with inhibitor we demonstrated that preincubation enhances inhibitor binding to wild type viruses, but has minimal effect on inhibitor binding to many viruses with reduced susceptibility to the NAIs, known to have lost the slow binding phenotype. Importantly, loss of slow binding is only seen for the drug to which the virus has reduced susceptibility. For example while we see loss of slow binding of both oseltamivir and peramivir to the H274Y virus, this has no impact on the binding kinetics of zanamivir to which the virus remains sensitive. Conversely we see loss of slow binding only of zanamivir and peramivir to the E119G virus, which has reduced susceptibility to both zanamivir and peramivir, but is sensitive to oseltamivir.

We also identified three wild type viruses with an intermediate phenotype, with similar final IC_50_s with or without preincubation, contrary to what is expected if they are slow binding (B/Perth/211/01, Indonesian Clade 2 H5N1 and A/Swine/Shepparton/2009 pandemic H1N1). Unlike the mutant viruses where the IC_50_s did not change much during the reactions, these viruses showed more than a 10-fold increase in IC_50_ in the preincubation reaction. This was much greater than for other wild type viruses. Interestingly similar phenotypes with greater than 10-fold increases in IC_50_s after preincubation were also seen with zanamivir and peramivir binding to the B/Perth/211/01 D197E mutant, thus suggesting these three wild type viruses have a low level of resistance. Dissociation studies of inhibitors are traditionally carried out by incubating virus or NA with excess inhibitor, removing unbound inhibitor by the use of a column, then following the enzyme activity [6], [22]–[24]. Our IC_50_ kinetics assays with the preincubation step indicate that despite the continued presence of inhibitor in the assay mixture, some dissociation occurs upon addition of substrate. Interestingly different relative dissociation rates can be distinguished between different inhibitors and different viruses. Also surprising was that without preincubation we saw rapid dissociation for DANA for all viruses and for most of the mutants with the drug to which they had reduced susceptibility. A possible explanation for this might be that while the inhibitor and substrate both bind rapidly, due to the higher affinity of the inhibitors they initially bind faster than the substrate. Then due to the higher substrate concentration this gradually displaces the inhibitor, leading to an increase in IC_50_.

While “slow” or “fast” binding are phenotypic markers of NA behaviour it is obvious that the final 60 min IC_50_s for the mutant viruses either with or without preincubation are similar. We have therefore done a semi-quantitative comparison. In general for all mutant viruses (except the NWS/G70C) the differences in the 60 min IC_50_s between no preincubation and preincubation were less than 2-fold for zanamivir, less than 1.5-fold for oseltamivir, and less than 2-fold for peramivir. Although the differences for the 60 min IC_50_s between the no preincubation and preincubation for NWS/G70C N9 were less than for other viruses, indicating that inhibitor binding is more rapid, there was still a larger difference for the wild type compared to the mutant virus.

It is well established that influenza B viruses are less sensitive to oseltamivir in enzyme assays [25]. There are also reports of lower clinical efficacy of oseltamivir in children infected with influenza B, compared to influenza A [26]. We have recently shown that the structural rearrangements of the E276, necessary for high affinity binding for oseltamivir do not appear to occur in influenza B. This is consistent with lack of slow binding and higher IC_50_s seen for influenza B viruses [11]. Similarly the faster binding of oseltamivir to the clade 2 H5N1 virus correlates with the higher IC_50_ [19]. Structurally this correlates with an H252Y difference between clade 1 and clade 2 NAs, which also prevents full rotation of the E276 [27] in the clade 2 virus. Hence the difference in binding kinetics is consistent with the known reduced susceptibility of both of the influenza B and clade 2 H5N1 wild type viruses to oseltamivir. While the apo structure of the pandemic N1 NA is known [28] the structure of the complex with oseltamivir has not yet been published. Since structural changes occur upon binding oseltamivir we cannot predict if there is any structural correlation to its apparent faster binding/dissociation to the pandemic H1N1 NA.

These results also demonstrate that there are factors other than the displacement of a water molecule by the guanidino group of zanamivir, or the rotation of the E276 for oseltamivir and peramivir that lead to slow binding of the NA inhibitors. The D197E mutation in the B/Perth/211/01 virus leads to altered interactions of the R152 with the N-acetyl group on the inhibitors, having no impact in the region of the guanidinium group or the rotation of the E276 [11] yet it leads to loss of slow binding. Characterisation of different mutants will help provide more understanding of factors involved in the slow binding of the NA inhibitors and what are the key interactions needed for high affinity binding other than those already identified. This information may be useful for design of new inhibitors.

There are other factors which are known to affect the IC_50_ and fold resistance of a mutant, such as use of the fluorescent or chemiluminescent assay and concentration of substrate [14], [25]. All inhibitors appeared to dissociate after preincubation with inhibitor on the addition of substrate, however there were different rates of dissociation for different viruses and inhibitors. Oseltamivir dissociated faster in many wild type viruses compared to the other two drugs, whereas dissociation of peramivir was very slow, consistent with observations by the classical methods demonstrating slow dissociation [22]–[24]. Hence longer reaction times will result in greater increases in the oseltamivir IC_50_s compared to increases in the IC_50_s of the other inhibitors. Because there is less change in the reaction rates with the mutant viruses their IC_50_ and *K*_i_ values were not as affected by the incubation or reaction times. However, our results here and previously published [11] clearly demonstrate that incubation time is an important factor which can affect both the IC_50_ and *K*_i_ differently for wild type and mutant viruses. Furthermore different incubation times in different laboratories would affect comparisons of the fold difference of wild type and mutant viruses. Understanding which variables affect the IC_50_s and *K*_i_s could ultimately lead to a better understanding of what is needed for a standardized assay to facilitate direct comparisons of isolates between different laboratories.

## Supporting Information

Table S1 Changes in IC_50_ (nM) between 10 and 60 min with or without preincubation^a^.
(DOC)
