# Supplementary material for: Real Time Enzyme Inhibition Assays Provide Insights into Differences in Binding of Neuraminidase Inhibitors to Wild Type and Mutant Influenza Viruses
Source: PLoS One. 2011 Aug 17;6(8):e23627. doi: 10.1371/journal.pone.0023627 (PMC3157426; doi:10.1371/journal.pone.0023627)
Supplement: Table S1 — Changes in IC50 (nM) between 10 and 60 min with or without preincubationa. (DOC) [file pone.0023627.s001.doc]

**Supplementary Table 1**

**Changes in IC_50_ (nM) between 10 and 60 min with or without preincubation^a^**

|  | **Time min** | **B/Perth wt^b^** | **B/Perth D197E** | **Miss’piH1N1 wt** | **Miss’pi H1N1 H274Y** | **Fukui H3N2 wt** | **Fukui H3N2 E119V** | **G70C H1N9 wt** | **G70C H1N9 E119G** | **G70C H1N9 R292K** | **Swine pH1N1wt** | **Avian H5N1 Clade1** | **Avian H5N1 Clade2** |
| --- | --- | --- | --- | --- | --- | --- | --- | --- | --- | --- | --- | --- | --- |
| **Zanamivir No pre** | 10 | 760 | 862 | 18.1 | 33.0 | 239 | 298 | 27.0 | 373 | 53.0 | 29.5 | 59.1 | 36.9 |
|  | 60.0 | 167 | 434 | 7.8 | 6.0 | 32.4 | 51.9 | 5.7 | 816 | 110 | 5.8 | 12.0 | 11.2 |
|  | **10'/60** | **4.5** | **2.0** | **2.3** | **5.5** | **7.4** | **5.7** | **4.7** | **0.5** | **0.5** | **5.1** | **4.9** | **3.3** |
| **Oseltamivir No pre** | 10 | 443 | 749 | 12.2 | 1905 | 30.4 | 178 | 18.1 | 13.9 | 21497 | 15.8 | 22.8 | 28.3 |
|  | 60.0 | 144 | 660 | 7.3 | 2353 | 5.3 | 208 | 4.0 | 5.4 | 30139 | 6.8 | 4.1 | 21.4 |
|  | **10'/60** | **3.1** | **1.1** | **1.7** | **0.8** | **5.7** | **0.9** | **4.5** | **2.6** | **0.7** | **2.3** | **5.5** | **1.3** |
| **Peramivir No pre** | 10 | 259 | 186 | 20.2 | 130 | 103 | 154 | 6.4 | 12.4 | 160 | 10.1 | 40.9 | 37.7 |
|  | 60.0 | 27.8 | 75 | 3.1 | 196 | 11.9 | 21.4 | 1.2 | 14.6 | 261 | 2.0 | 6.2 | 5.4 |
|  | **10'/60** | **9.3** | **2.5** | **6.5** | **0.7** | **8.7** | **7.2** | **5.3** | **0.9** | **0.6** | **5.1** | **6.6** | **7.0** |
| **Zanamivir 30’ Pre** | 10 | 2.1 | 5.5 | 0.9 | 1.7 | 2.9 | 1.8 | 1.6 | 332 | 62.4 | 0.5 | 0.6 | 0.6 |
|  | 60.0 | 8.9 | 258 | 1.9 | 2.2 | 3.8 | 3.4 | 2.7 | 678 | 94.8 | 1.4 | 2.5 | 1.3 |
|  | **60'/10** | **4.2** | **46.8** | **2.1** | **1.3** | **1.3** | **1.9** | **1.7** | **2.0** | **1.5** | **2.5** | **4.5** | **2.2** |
| **Oseltamivir 30’ Pre** | 10 | 7.9 | 274.8 | 0.5 | 1586 | 0.5 | 122 | 1.1 | 0.6 | 17297 | 0.5 | 0.2 | 0.9 |
|  | 60.0 | 104 | 708.0 | 3.1 | 2440 | 1.7 | 260 | 2.8 | 2.9 | 24692 | 5.1 | 0.6 | 19.6 |
|  | **60'/10** | **13.1** | **2.6** | **5.9** | **1.5** | **3.7** | **2.1** | **2.6** | **4.7** | **1.4** | **10.4** | **2.9** | **22.5** |
| **Peramivir 30’Pre** | 10 | 1.9 | 3.9 | 0.3 | 100 | 1.0 | 1.4 | 0.4 | 3.5 | 127 | 0.2 | 0.2 | 0.4 |
|  | 60.0 | 2.8 | 41.5 | 0.4 | 153 | 1.3 | 2.1 | 0.4 | 13.3 | 260 | 0.3 | 0.3 | 0.6 |
|  | **60'/10** | **1.5** | **10.7** | **1.4** | **1.5** | **1.3** | **1.5** | **1.0** | **3.8** | **2.0** | **1.5** | **1.3** | **1.4** |

^a^IC_50_ values are the average of duplicate assays.

**^b^** Viruses , wt = wild type, mutants have specific mutation defined. B/Perth/211/01, A/Mississippi/03/01, A/Fukui/45/04, A/NWS/tern/Australia/G70C/75, pandemic H1N1 A/Swine/Shepparton/2009, avian H5N1 Vietnam clade 1, avian H5N1 Indonesian clade 2.
